# Supplementary material for: Serum GlycA Level Is Elevated in Active Systemic Lupus Erythematosus and Correlates to Disease Activity and Lupus Nephritis Severity
Source: J Clin Med. 2020 Mar 31;9(4):970. doi: 10.3390/jcm9040970 (PMC7230647; doi:10.3390/jcm9040970)
Supplement: Supplementary file 1 [file jcm-09-00970-s001.pdf]

Supplementary Tables

Supplementary Table S1. Demographic and clinical parameters.

	Characteristics	Healthy Controls (n=20)	Non-Lupus KD (n =21)	Quiescent SLE (n = 39)	Active SLE (n =105)	Non-Proliferative LN (n = 10)	Proliferative LN (n = 26)
**Demographic charactericstics**	Age (median, IQR)	36, 28-42	48, 35-56	35, 28-47	32, 26-42	35, 28-38	32, 25-39
	Gender female (%)	80	38	95	94	90	88
	Ethnicity caucasian (%)	NA	NA	67	82	80	85
	BMI (median, IQR)	NA	23, 21-27	21, 20-23	23, 21-27	29, 26-31	23, 20-26
	Current smoking (%)	NA	19	5	16	40	28
**Clinical characteristics**	SLEDAI (median, IQR)	NA	NA	4, 2-4	8, 6-12	8, 4.5-13.5	12, 8-18
	CS daily dose (median, IQR) (mg)	NA	0, 0-0	6, 4-8	6, 4-10	5, 0-10	6, 4-20
	Hydroxychloroquine (%)	NA	0	95	84	60	77
	CRP (median, IQR mg/L)	NA	2.2, 1.0-5.7	1, 0.8-1.2	2.5, 1-6.3	3.5, 2.2-5.3	2.7, 1-5.8
**Lab values**	Serum albumin (median, IQR) (g/L)	NA	38, 32-41	41, 39-43	36, 33-38	34, 30-37	32, 28-36
	Serum creatinin (median, IQR) (µmol/L)	NA	172, 97-303	65, 56-83	68, 55-88	60, 55-67	73, 57-95
	eGFR (median, IQR) (mL/min/1.73m²)	NA	34, 18-111	87, 64-115	98, 63-120	110, 97-125	93, 73-117
	Neutrophil (median, IQR) (10^9/L)	NA	NA	NA	NA	3.2, 2.9-4.3	5.5, 2.6-7.4
	C3 (median, IQR) (g/L)	NA	NA	0.86, 0.78-0.91	0.72, 0.54-0.96	1.04, 0.54-1.26	0.63, 0.45-0.88
	C4 (median, IQR) (g/L)	NA	NA	0.15, 0.13-0.17	0.11, 0.06-0.19	0.22, 0.08-0.25	0.10, 0.04-0.20
	dsDNA antibody positivity (%)	NA	NA	90	90	60	91
	UPCR (median, IQR) (g/g)	NA	2, 0.47-5.90	0.30, 0.08-0.56	1.19, 0.62-2.49	1.29, 0.60-2.05	1.94, 1.05-4.39
	Hematuria (%)	NA	NA	NA	NA	66.6	70
	Leukocyturia (%)	NA	NA	NA	NA	55.5	60

Supplementary Table S2. Multivariate models.

Model	Variables							
	**GlycA**		**BMI**					
	OR	p	OR	p				
**GlycA + BMI**	14.9 [2.6-3230]	**2,02E-03**	0.12 [0.008-0.03]	**4,58E-04**				
					**eGFR**		**UPCR**	
					OR	p	OR	p
**GlycA + BMI + eGFR + UPCR**	2801 [15.8-2.89e7	**6,42E-04**	0.685 [0.42-0.89]	**1,97E-03**	0.98 [0.92-1.04]	4,44E-01	1.04 [0.76-1.42]	7,88E-01
					**C3**		**C4**	
					OR	p	OR	p
**GlycA + BMI + C3 + C4**	81.1 [3.0-1.5e5]	**2,79E-03**	0.12 [0.001-1.3]	**9,34E-02**	1.1 [0.03-56.9]	9,47E-01	0.52 [0.003-83.6]	7,88E-01
					**dsDNA**			
					OR	p		
**GlycA + BMI + dsDNA**	209 [7-1.2e6]	**2,28E-05**	0.24 [0.008-1.48]	1,31E-01	77.3 [0.51-3.0e7]	**9,81E-02**		
					**Daily Corticosteroid Dosage**			
					OR	p		
**GlycA + BMI + CortDosage**	17.4 [2.4-567]	**1,05E-03**	0.13 [0.008-0.6]	**5,48E-03**	1.38 [0.45-5.37]	5,79E-01		
					**Serum Triglycerides**			
					OR	p		
**GlycA + BMI + Serum TG**	49.5 [3-7.2e3]	**2,83E-03**	0.084 [0.003-0.46]	**1,24E-03**	0.29 [0.15-3.0]	2,99E-01		
					**Neutrophil Count**			
					OR	p		
**GlycA + BMI + Neutrophil Count**	13.314 [2-398]	**3,60E-03**	0.102 [0.004-0.55]	**3,51E-03**	2.23 [0.32-26.0]	4,42E-01		

Supplementary Figures:

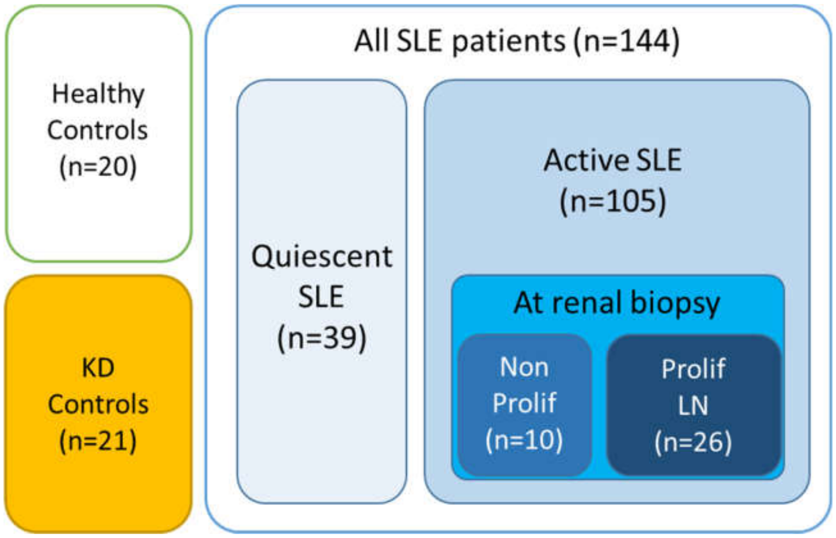

Supplementary Figure S1. Sample overview.

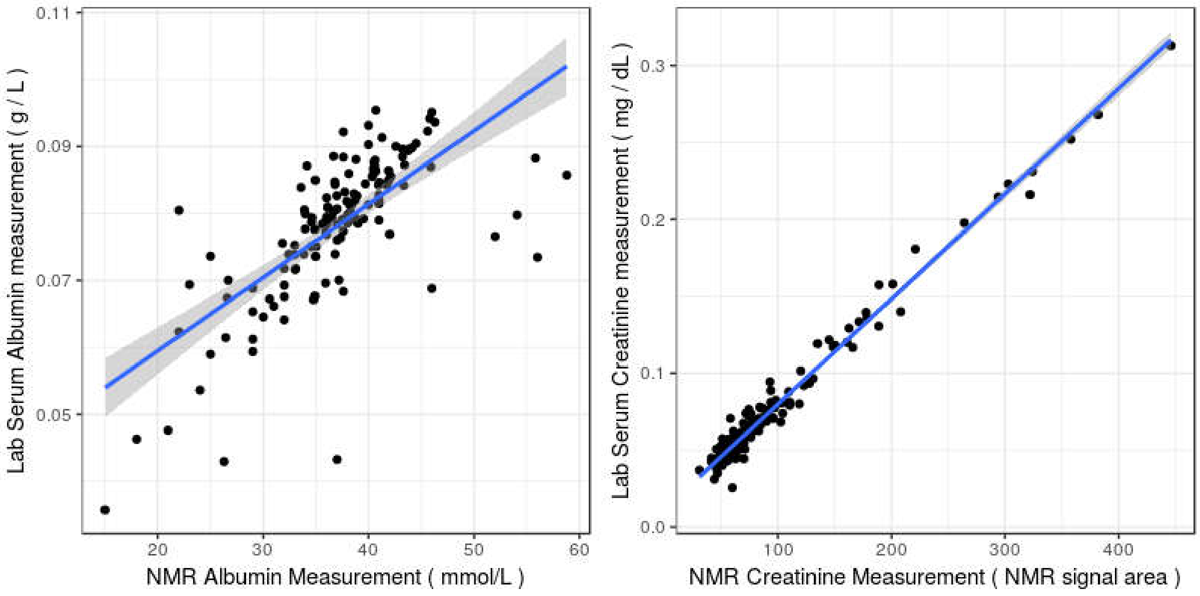

Supplementary Figure S2. NMR measurements and standard laboratory tests correlate very well for both serum albumin and creatinine levels (ϱ = 0.74 and ϱ = 0.94, respectively, with *p*-values<10-8).
